# Sexual dimorphism in benign adrenocortical tumours

**DOI:** 10.1093/ejendo/lvaf088

**Published:** 2025-04-29

**Authors:** Onnicha Suntornlohanakul, Cristina L Ronchi, Wiebke Arlt, Alessandro Prete

**Affiliations:** Endocrinology and Metabolism Unit, Division of Internal Medicine, Faculty of Medicine, Prince of Songkla University, Songkhla 90110, Thailand; Department of Metabolism and Systems Science, College of Medicine and Health, University of Birmingham, Birmingham B152TT, United Kingdom; Department of Metabolism and Systems Science, College of Medicine and Health, University of Birmingham, Birmingham B152TT, United Kingdom; Centre for Endocrinology, Diabetes and Metabolism, Birmingham Health Partners, Birmingham B152TT, United Kingdom; Department of Endocrinology, Queen Elizabeth Hospital Birmingham, University Hospitals Birmingham NHS Foundation Trust, Birmingham B152GW, United Kingdom; Medical Research Council Laboratory of Medical Sciences, London W120NN, United Kingdom; Institute of Clinical Sciences, Imperial College London, London SW72AZ, United Kingdom; Department of Metabolism and Systems Science, College of Medicine and Health, University of Birmingham, Birmingham B152TT, United Kingdom; Centre for Endocrinology, Diabetes and Metabolism, Birmingham Health Partners, Birmingham B152TT, United Kingdom; Department of Endocrinology, Queen Elizabeth Hospital Birmingham, University Hospitals Birmingham NHS Foundation Trust, Birmingham B152GW, United Kingdom; National Institute for Health and Care Research (NIHR) Birmingham Biomedical Research Centre, University of Birmingham, University Hospitals Birmingham NHS Foundation Trust, Birmingham B152GW, United Kingdom

**Keywords:** sexual dimorphism, adrenal tumour, Cushing's syndrome, mild autonomous cortisol secretion, primary aldosteronism

## Abstract

Benign adrenocortical tumours are the most common adrenal neoplasms. Evidence over the past few decades has highlighted sex differences in their prevalence, clinical characteristics, and treatment outcomes. Cortisol-producing adenomas causing either Cushing's syndrome, particularly those with *PRKACA* or *GNAS* somatic mutations associated with a more severe phenotype, or mild autonomous cortisol secretion (MACS) are more commonly observed in women. The mechanisms underpinning this sexual dimorphism remain to be fully elucidated. Studies in mice have revealed a protective role of androgens in males, leading to a decelerated growth rate of adrenocortical cells. Furthermore, evidence from human adrenal tumour tissue suggests that oestrogen, progesterone, and luteinising hormone/choriogonadotropin signalling in the adrenal cortex may play a role in adrenal tumourigenesis and steroid production. Clinically, this is supported by the increased incidence of cortisol-producing adrenocortical adenomas or nodular hyperplasia during puberty, pregnancy, and menopause. Notably, women with MACS seem to be more vulnerable to the harmful effects of cortisol excess and carry a higher mortality risk than men. Women with aldosterone-producing adenomas have a higher prevalence of somatic *KCNJ5* mutations than men, and patients harbouring these mutations are likely to have more favourable clinical outcomes after adrenalectomy. In this review, we summarise the possible mechanisms behind the sexual dimorphism of benign adrenocortical tumours and provide an up-to-date overview of the sex-specific differences in their prevalence, clinical presentation, and outcomes, focusing on cortisol and aldosterone excess. Considering sexual dimorphism is crucial to guide diagnosis and management, and to counsel these patients for optimised care.

## Graphical Abstract

**Graphical Abstract:**
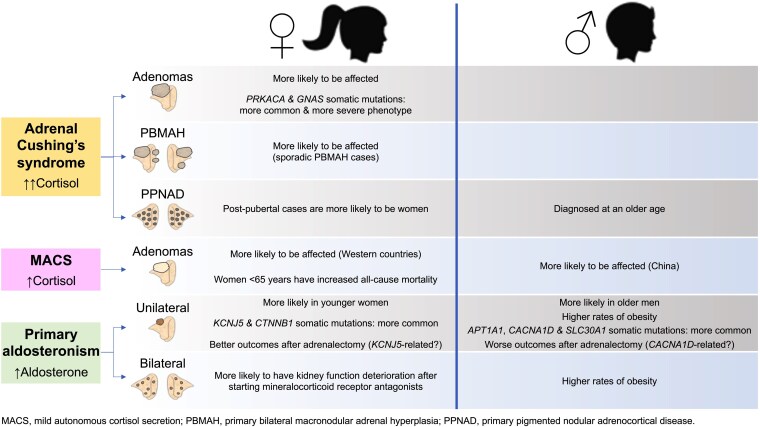

**Significance**

Benign adrenocortical tumours are among the most common human neoplasms, yet their pathophysiology remains incompletely understood. This review highlights the emerging evidence of sexual dimorphism in these tumours, revealing key differences in prevalence, clinical presentation, and treatment outcomes between men and women. Recognising these sex differences is critical for optimising diagnosis and management strategies in endocrine practice, ensuring more personalised and effective care for patients with benign adrenocortical tumours.

## Introduction

Adrenal tumours are found in 1.4%-7% of adults undergoing abdominal imaging,^1,2^ with a 10-fold increase in incidence over the last 3 decades, mainly driven by the rise of cross-sectional imaging use.^3^ Benign adrenal tumours, mostly adrenocortical adenomas, account for ∼70%-95% of newly diagnosed adrenal tumour cases.^4,5^ Overall, adrenal tumours affect men and women almost equally, with a slight female predominance (45% and 55%, respectively);^3,5^ however, benign adrenocortical tumours (BATs) are observed more frequently in women.^6,7^ Sex chromosomes may contribute to sexual dimorphism through mechanisms including X-chromosome inactivation escape and epigenetic regulation;^8,9^ however, evidence linking sex chromosomes to sex-related differences in BATs is lacking. Instead, hormonal factors appear to be the primary drivers.

Recent studies highlighted sex differences in BAT prevalence, clinical manifestations, and outcomes impacting diagnosis and patient management. In this review, we discuss sexual dimorphism in BATs, focusing on putative contributing mechanisms linked to adrenal gland development, proliferation, and tumourigenesis. Differences in prevalence, clinical manifestations, and outcomes of BATs with different hormone secretion are also discussed.

## Sex differences in adrenal development, proliferation, and tumourigenesis

### Adrenal cortex development

The adult human adrenal cortex is divided into 3 zones. The zona glomerulosa (ZG), which produces mineralocorticoids, is mainly regulated by the renin-angiotensin-aldosterone system. The zona fasciculata (ZF) and zona reticularis (ZR), which produce glucocorticoids and androgen precursors, respectively, are controlled by the hypothalamic-pituitary-adrenal axis (HPAA).

**Figure 1.**
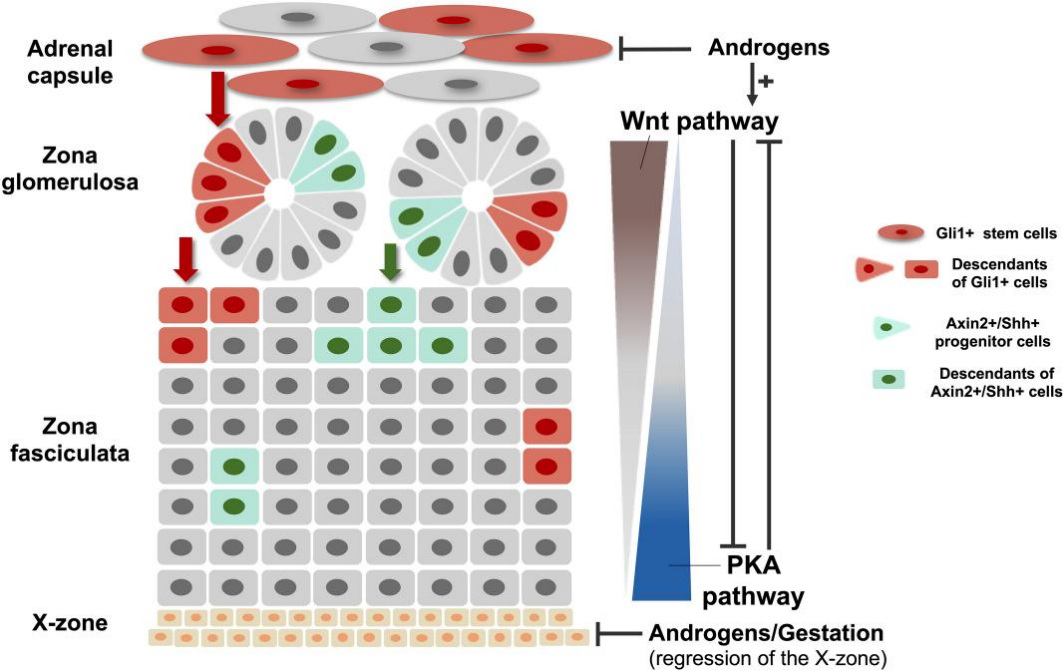
Adrenal cortex renewal from mouse models. The adrenal gland continuously regenerates itself by stem cell proliferation and migration from the capsule or subcapsular zona glomerulosa to the inner zone (centripetal migration model). In female mice, the capsular Gli1+ stem cells and the Axin2+/Shh+ progenitor cells are responsible for this process, whereas androgens inhibit the proliferation and migration of the Gli1+ stem cells in males. Other cells, including WT1-positive and nestin-positive progenitor cells, are also involved in replenishing the adrenal gland (not shown). As regenerated cells migrate to the inner zone, they mature into zona glomerulosa and fasciculata cells. Cell migration and zonal differentiation are influenced by the interaction between the Wnt and protein kinase A (PKA) signalling pathways. The Wnt pathway stimulates zona glomerulosa cell differentiation (with a lessening activation gradient from the zona glomerulosa to the zona fasciculata), while PKA signalling promotes the differentiation into zona fasciculata cells (with a lessening activation gradient from the zona fasciculata to the zona glomerulosa). The fate of adrenocortical cells is partially determined by the interplay between these 2 pathways.

Adrenal gland development begins with the formation of the adreno-gonadal primordium at 28-30 days post-conception in humans (embryonic day [E] 9.0 in mice), later separating into the adrenal primordium and gonadal primordium, which develop into an adrenal foetal zone and gonads, respectively. The neural crest cells then migrate to the adrenal primordium to form the adrenal medulla, and the encapsulation—the final step of foetal adrenal gland development—occurs at around 52 days post-conception in humans and ∼E14 in mice. At eighth-week gestation (∼E16 in mice), the definitive zone starts appearing between the capsule and foetal cortex. After birth, the definitive zone differentiates into ZG and ZF, whereas the foetal zone regresses. The ZR begins to form and produce adrenal androgens at 6-9 years of age (adrenarche).^10-12^ In mice, the residual foetal zone (X-zone) regresses when male mice enter puberty and female mice get their first pregnancy; the early X-zone regression in males is regulated by androgens.^13,14^ After development, the adrenal cortex must continually regenerate to maintain normal size and function. The centripetal model, which describes that the capsular and subcapsular stem cells migrate to replace the adrenal tissue in each zone, is the most widely accepted (Figure 1), based on experimental evidence.^15^

### Sexual dimorphism in adrenal gland renewal—the effect of androgens

Mouse models demonstrated sex differences in adrenal gland anatomy, development, and renewal.^7,16-18^ Female mice have greater adrenal cortex and medulla weights compared to males; the cortex (∼80% of the gland) primarily contributes to these sex differences, mainly driven by the higher ZF growth rates in females during puberty.^19^ Moreover, female mice have additional adrenocortical stem cells and the adrenocortical turnover rate is 3 times higher than in males.^20^ In both sexes, subcapsular Axin2+/Shh+ progenitor cells can proliferate and differentiate into steroidogenic cells; on the other hand, the capsular Gli1+ stem cells only undergo adrenocortical differentiation in females during normal homeostasis (Figure 1). However, the capsular Gli1+ stem cells can contribute to adrenocortical regeneration in males after atrophy induced by high-dose dexamethasone.^21^ Androgens appear to drive the sexual dimorphism of adrenocortical renewal, as sex-specific differences disappear after gonadectomy of male mice and dihydrotestosterone treatment of female mice.^20^ Furthermore, the cAMP-protein kinase A (PKA) pathway, which is responsible for stem cell recruitment and differentiation in the adrenal, is inhibited by androgens in mice, possibly by sustaining the Wnt/β-catenin signalling pathway.^22^ Whether mouse models are transferable human physiology is, however, still an open question.

### Impact of oestrogen and progesterone signalling on the adrenal cortex

**Figure 2.**
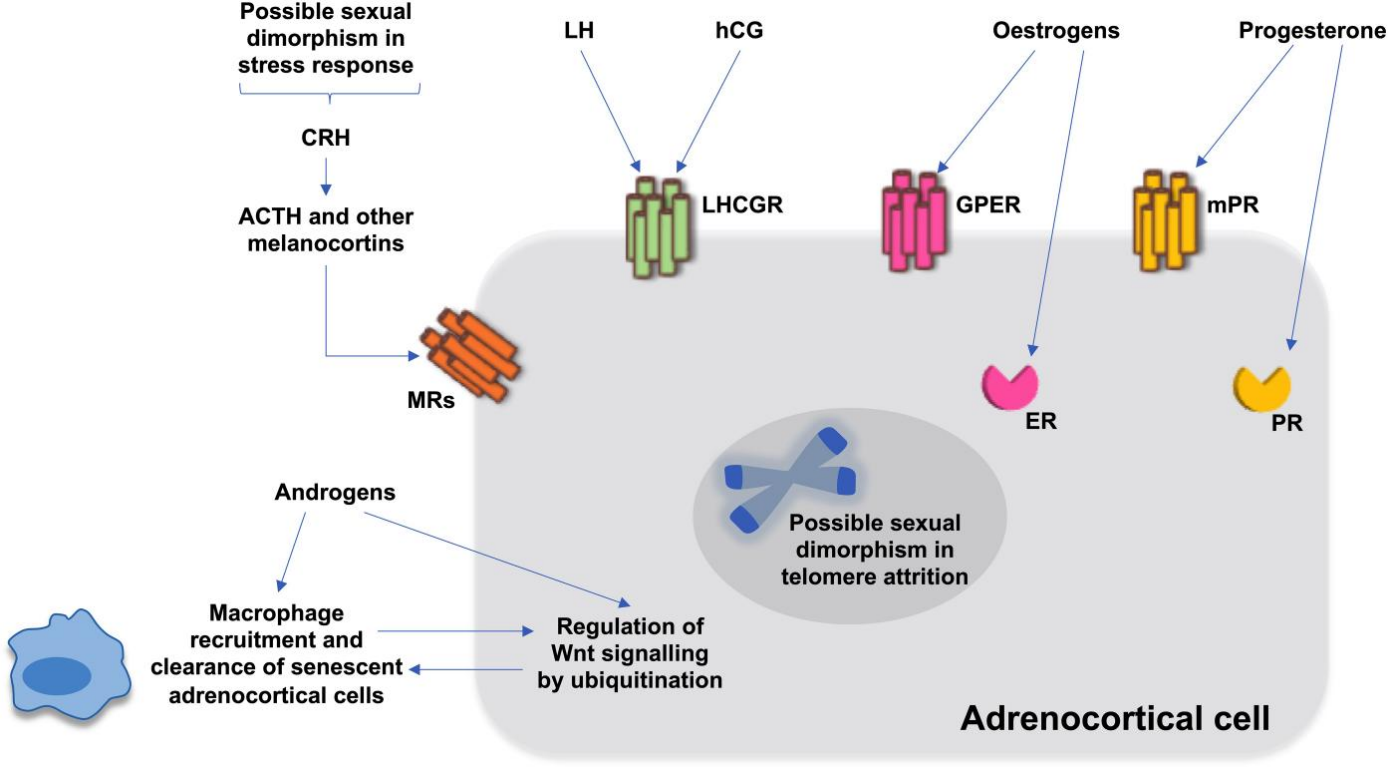
Putative mechanisms contributing to the sexual dimorphism of adrenocortical tumourigenesis. Factors potentially involved in the sexual dimorphism of adrenocortical tumours include (1) oestrogen, progesterone, and luteinizing hormone/choriogonadotropin signalling, (2) differences in the intra-adrenal immunological milieu (described in mouse models of adrenocortical carcinoma only), (3) possible differences in the stress response, and (4) differences in telomere attrition between sexes. The effects of androgens on adrenal gland formation, proliferation, and renewal are shown in Figure 1. Abbreviations: ACTH, adrenocorticotropic hormone; CRH, corticotropin-releasing hormone; ER, nuclear oestrogen receptor; GPER, G protein-coupled oestrogen receptor; hCG, human chorionic gonadotropin; LH, luteinising hormone; LHCGR, luteinising hormone/choriogonadotropin receptor; mPR, membrane progesterone receptor; MR, melanocortin receptor; PR, nuclear progesterone receptor.

Oestrogen receptors (ER) and progesterone receptors (PR) are identified in the normal adrenal cortex and tumours and may play a role in tumourigenesis and steroidogenesis (Figure 2).^23-25^ There are 2 main types of ER: the nuclear ERα and ERβ, and the G protein-coupled ER (GPER). A study found immunoreactivity of ERα in the definitive zone, but not the foetal zone, of the human foetal adrenal gland.^25^ Gene set enrichment analysis of single-nuclei transcriptomes in adult human normal adrenal glands also showed high expression of oestrogen signalling in the ZR.^26^ In female mice, oestrogen deficiency causes arrest of adrenal cell proliferation resulting from telomerase inhibition, which is reversed by oestrogen replacement.^27^ High ERβ and PR mRNA levels were found in both normal adrenocortical tissues and BATs.^24^ Therefore, it is intriguing to speculate an involvement of oestrogen and progesterone signalling in adrenal tumourigenesis in women, although mechanistic studies are lacking.^24,25,28^ Furthermore, this does not explain the higher prevalence of BATs in post-menopausal women, where oestrogen and progesterone levels are low. Oestrogen signalling also plays a role in physiological aldosterone production and primary aldosteronism (PA). Under physiological conditions, oestrogens suppress aldosterone production by binding to ERβ in ZG cells. However, in aldosterone-producing adenomas (APAs), oestrogens stimulate aldosterone secretion through GPER-1, predominantly expressed in adenoma cells.^29^

### Impact of gonadotropin signalling on the adrenal cortex

The luteinising hormone/choriogonadotropin receptor (LHCGR), typically found in gonads, is also expressed in the normal adrenal cortex and has been identified in adrenocortical tumours and primary bilateral macronodular adrenal hyperplasia (PBMAH).^30-34^ Adrenal cells expressing LHCGR also express the cytochrome P450 side chain cleavage enzyme, supporting they can be steroidogenic.^30^ Chronic LH or hCG elevation during menopause or pregnancy may be a contributing factor to adrenocortical hyperplasia and tumourigenesis (Figure 2).^35-37^ For example, several cases of adrenal Cushing's syndrome (CS) potentially attributed to LHCGR stimulation have been reported in PBMAH or adrenocortical adenomas diagnosed during pregnancy.^32,34,38-40^ Some cases experienced remission postpartum and relapsed during menopause. A positive correlation between LH levels and insulin resistance was shown in post-menopausal women with BATs but not in healthy controls matched for age, body mass index (BMI), LH levels, and menopause duration. This suggests that insulin may promote cell proliferation by activating the IGF-1 receptor in normal and neoplastic adrenal cells.^41,42^ In mice, inhibin and activin signalling is involved in adrenal tumourigenesis in the context of chronic LH elevation.^43,44^

### Sexual dimorphism in the immune system remodelling within the adrenal gland

Sex differences in intra-adrenal immunity have been studied only in mouse adrenocortical carcinoma (ACC) models. *ZNRF3* is a tumour suppressor gene that encodes a membrane E3 ubiquitin ligase, which inhibits the Wnt/β-catenin signalling pathway, known to play a role in adrenal tumourigenesis. Two studies have investigated the immune response to adrenal tumourigenesis in *ZNRF3* knockout mouse models.^45,46^ Wilmouth Jr et al.^45^ demonstrated that *ZNRF3* knockout female mice developed ACC after 18 months, while male mice experienced regression of the adrenal glands. This sex-specific outcome was influenced by androgens, which triggered a senescence-associated secretory phenotype in males, characterised by activation of highly phagocytic macrophages, clearing out pre-neoplastic cells and preventing ACC development (Figure 2). In contrast, macrophage recruitment in female mice was delayed and suppressed, allowing progression to ACC. Another study supported these findings, reporting that 62% of *ZNRF3* knockout female mice developed ACC, compared to only 8% of male mice;^46^ these results were attributed to a senescence-associated secretory phenotype. This model also revealed that dendritic cells are essential in preventing ACC development. However, whether these findings can apply to BATs or humans has yet to be determined. Single-nucleus and spatial transcriptome analysis showed that adrenocortical adenomas exhibit a lower number of lymphoid and myeloid cells compared to normal human adrenal glands. Notably, the myeloid cells in cortisol-producing adrenocortical adenomas (CPAs) associated with CS appears to be diminished relative to both normal adrenals and non-functioning adrenal adenomas, potentially due to the inhibitory effects of glucocorticoids on tumour-immune infiltration.^26^

### Sexual dimorphism in the hypothalamic-pituitary-adrenal axis response to stress

Sexual dimorphism in the HPAA stress response may contribute to adrenal tumourigenesis. Animal models have shown sex differences in the HPAA stress response,^47-51^ while evidence in humans remains inconclusive, likely due to confounding factors such as age, menstrual cycle variations in women, and stress type and duration.^52^ Compared to males, female rats had higher levels of corticosterone and adrenocorticotropic hormone (ACTH) after exposure to acute stressors, coupled with increased mRNA expression of corticotropin-releasing hormone and the ACTH precursor proopiomelanocortin (POMC).^47-50^ POMC is cleaved to pro-γ melanocyte-stimulating hormone (pro-γ MSH), which has a proliferative effect on the adrenal cortex.^53-55^

### Role of telomere length in sexual dimorphism

Nonaka et al.^56^ found that adrenal weight declined with age in older men, while it remained constant in age-matched women. This was attributed to ZF depletion, as the ZF accounts for most of the adrenal cortex weight. In line with this, the authors found that the telomere length—a regulator of cell proliferation closely linked to senescence^57^—was shorter in the ZF of older men, suggesting that the weight decline could be driven by telomere attrition (Figure 2).^56^ However, conflicting evidence from another study, reporting age-related ZF and adrenal cortex enlargement in both sexes, highlights the need for further research to understand the mechanisms underpinning sex-specific differences in adrenal ageing.^58^

## Sexual dimorphism in adrenal incidentaloma prevalence

Adrenal incidentalomas, ie, adrenal tumours discovered during investigations carried out for reasons unrelated to adrenal disease, are found in 1.4%-7% of adults with prevalence varying based on the population studied, the discovery mode, and the data source.^1,2^ Several studies from Western populations showed a higher prevalence of incidentalomas in women, with a female-to-male ratio of 1.4-1.9:1,^3,6,59-61^ but this was not consistently shown in Asian populations—where 51%-57% of incidentaloma cases are men—and in autopsy studies from various populations.^2,62-66^ The reason for the discrepancies among these studies is unclear. Women were found to have higher medical care service utilisation in some studies,^67-69^ and as such may receive more cross-sectional imaging of the abdomen, but this was not observed consistently.^70,71^

## Sexual dimorphism in adrenal Cushing's syndrome

Adrenal CS describes ACTH-independent, clinically overt cortisol excess caused by adrenocortical adenomas, carcinomas, or hyperplasia. This section will focus on sex differences in benign causes of adrenal CS (Table 1).

**Table 1.** Sex differences in the prevalence and clinical characteristics of benign adrenocortical tumours.

		Sex distribution	Sex differences in clinical characteristics and outcomes
Adrenal Cushing's syndrome	Adrenocortical adenomas	Female-to-male ratio 4-13:1 Somatic *PRKACA* and *GNAS* mutations are much more common in females (female-to-male ratio 13:1).	Women ≥ 65 years may be more likely to be diagnosed incidentally. Patients with *PRKACA* or *GNAS* mutations have younger age and a more severe clinical Cushing phenotype.
	Primary bilateral macronodular adrenal hyperplasia	Female-to-male ratio 2-3:1 in sporadic, *ARMC5*-negative cases	All patients with food-dependent Cushing's syndrome and *KDM1A* mutations in published studies were women.
	Primary pigmented nodular adrenocortical disease (PPNAD)	Female-to-male ratio 0.5:1 in subjects < 12 years; 2-2.4:1 in subjects ≥ 12 years	Women with germline *PRKAR1A* mutations are more likely than men to be diagnosed with PPNAD after puberty and develop PPNAD at a younger age.
Mild autonomous cortisol secretion		Female-to-male ratio 0.7-2.2:1^a^	Women < 65 years carry a higher all-cause mortality risk than men.
Primary aldosteronism	Unilateral primary aldosteronism	Women < 50 years are more likely to have unilateral primary aldosteronism than older women. Somatic *KCNJ5* and *CTNNB1* mutations are more common in women (female-to-male ratio 1.6-4.4:1 and 2:1, respectively). Somatic *ATP1A1* and *CACNA1D* mutations are more common in men (female-to-male ratio ∼0.2:1 and ∼0.3:1, respectively). Somatic *SLC30A1* mutations have been reported exclusively in men.	Blood pressure control after adrenalectomy tends to be better in women. Patients who harbour *KCNJ5* mutations have better clinical outcomes after surgery. Patients who harbour *CACNA1D* mutations have worse clinical outcomes after surgery. Men have higher rates of obesity than women.
	Bilateral primary aldosteronism	Women ≥ 50 years have a higher likelihood of having bilateral primary aldosteronism than younger women.	Men have higher rates of obesity than women. Women with bilateral primary aldosteronism seem to have a higher cardiometabolic burden (obesity, diabetes, dyslipidaemia) than those with unilateral primary aldosteronism. Among patients with bilateral primary aldosteronism, women may be more likely than men to have deterioration of renal function after starting mineralocorticoid receptor antagonists.

^a^Most studies demonstrated a higher female-to-male ratio in patients with mild autonomous cortisol secretion (MACS), but 1 prospective study from a Chinese population showed that only 17 out of 40 patients with MACS were women (female-to-male ratio of 0.7).

### Adrenocortical adenomas

Cortisol-producing adrenocortical adenomas more frequently affect women (female-to-male ratio 4-13:1; Table 1) and are the most common aetiology of adrenal CS, accounting for approximately 25% of all adult CS cases^72-77^ and up to 44% of CS cases diagnosed during pregnancy.^78^ Age did not appear to have a substantial effect on the prevalence of adrenal CS in the ERCUSYN registry,^77^ although a smaller-scale study found that CPAs were the leading cause of CS in women > 65 years, who were often diagnosed incidentally.^79^ A study from South Korea found no significant sex differences in mortality in patients with adrenal CS.^80^ Currently, the reasons behind the predominance of CS due to adrenocortical adenoma in women remain unclear, but ER, PR, and LHCGR signalling may play a role, as discussed above. Furthermore, a notable sex difference is observed in the prevalence of somatic mutations within these tumours with the 2 most common mutations, *PRKACA* and *GNAS*, almost exclusively detected in women (86%-100% of mutated cases).^81-85^ Patients with these cAMP-related gene mutations are likely to experience a more severe form of CS and be diagnosed at a younger age. The mechanisms driving this sexual dimorphism are unknown.

### Primary bilateral macronodular adrenal hyperplasia

Primary bilateral macronodular adrenal hyperplasia is a rare cause of CS and is associated with germline *ARMC5* mutations in 20%-80%, with frequency increasing in the presence of a positive family history, larger adrenals or more nodules, and higher degree of cortisol excess.^86,87^ In sporadic cases, PBMAH is more prevalent in women with a female-to-male ratio of 2-3:1 (Table 1), although no sex differences are observed in *ARMC5*-positive cases.^87,88^ Recently, germline *KDM1A* mutations have been described in very rare food-dependent CS in PBMAH cases, and all identified patients were women.^89^ Notably, the first case of primary unilateral macronodular adrenal hyperplasia (PUMAH) with concomitant cortisol and androgen excess was described, again with germline *KDM1A* mutations present; the affected patient was a woman presenting during pregnancy.^90^

### Primary pigmented nodular adrenocortical disease

Primary pigmented nodular adrenocortical disease (PPNAD), a form of bilateral micronodular adrenal hyperplasia, is associated with Carney Complex (CNC), a rare autosomal dominant disorder characterised by endocrine and non-endocrine manifestations, including cardiac myxomas, lentigines, and pituitary adenomas. However, PPNAD can also occur without other features of CNC (isolated PPNAD). Most CNC-related and isolated PPNAD cases are caused by germline inactivating mutation of the gene encoding the regulatory subunit of the protein kinase A (*PRKAR1A*).^7^

In a large cohort of patients with germline *PRKAR1A* mutations and/or CNC phenotype, PPNAD prevalence was similar between sexes during childhood; however, after puberty, women were more likely to be diagnosed (female-to-male ratio 2-2.4:1 in patients aged ≥12 years), suggesting a possible influence of sex hormones.^91^ Women with PPNAD were also diagnosed at a significantly younger age (median 30 years vs 46 in men):^91^ At the age of 40, more than 70% of women with germline *PRKAR1A* mutations developed PPNAD, compared to only 45% of men.^72,91^ A study in mice demonstrated that *PRKAR1A* deletion led to constitutive PKA activation, resulting in the development of adrenal hyperplasia resembling PPNAD and causing CS in young females.^22,92^ In contrast, male mice displayed less severe clinical phenotypes, suggesting a possible protective effect of androgens, involving the Wnt signalling pathway and counteracting the PKA cascade.

## Sexual dimorphism in adrenocortical adenomas associated with mild autonomous cortisol secretion

Mild autonomous cortisol secretion (MACS) is the most common hormonal abnormality in patients with adrenal incidentalomas, with a prevalence of 20%-50%.^2-4,6,93,94^ Mild autonomous cortisol secretion is defined by serum cortisol > 50 nmol/L after administration of 1 mg dexamethasone overnight, without clinical signs of CS. Studies from Western countries showed higher MACS prevalence in women (66%-69%, female-to-male ratio up to 2.2:1),^4,6,61,95^ while in a study from China, men were more frequently affected (57.5%; female-to-male ratio 0.7:1).^2^ Reasons for this discrepancy are unclear but they may be due to ethnic differences, different study designs, and a relatively low rate of hormonal testing in the study from China.^96^

Over 80% of women with MACS from Western countries are post-menopausal (median age at diagnosis 64 years).^6^ As discussed above, persistent LH stimulation of the adrenal cortex can potentially link menopause to adrenocortical tumourigenesis. Similarly, high levels of LH may promote glucocorticoid production: A positive correlation between LH levels, serum morning cortisol, and 24-h urinary-free cortisol levels has been observed in post-menopausal women.^97,98^

Patients with MACS have higher cardiometabolic risk, cardiovascular events and mortality compared to non-functioning adenomas.^6,61,95,99-101^ Interestingly, a large retrospective multicentre cohort study found that women under the age of 65 had the highest risk of all-cause mortality (HR 4.39 [95% CI: 1.93-9.96] vs non-functioning adenomas);^95^ in contrast, the risk for men of similar age was not increased. Other smaller observational studies also found that younger women with MACS may be more vulnerable to the harmful effects of excess cortisol, leading to early onset of comorbidities such as dysglycaemia and hypertension compared to men.^100,102^

There are several hypotheses on why women seem to be more adversely affected by MACS. The adrenal-specific enzyme cytochrome P450 11β-hydroxylase (CYP11B1), converting 11-deoxycortisol to cortisol, is also a key regulator of adrenal-derived 11-oxygenated androgen production. Therefore, 11-oxygenated androgens are likely to increase in line with glucocorticoid excess in the context of MACS and adrenal CS, with some cases reported,^103,104^ and, similarly to polycystic ovarian syndrome, could correlate with an unfavourable cardiometabolic risk profile in women.^105^ Sex disparity in managing cardiovascular risk factors might also lead to sex-specific differences in cardiovascular morbidity: Men may receive more aggressive treatment for cardiovascular risk factors than women.^106^ Lastly, gene expression in response to synthetic glucocorticoid administration differs between male and female rats, suggesting that glucocorticoid action in target organs may be sexually dimorphic, leading to different outcomes of exposure.^107,108^

## Sexual dimorphism in PA

Primary aldosteronism is a condition of renin-independent autonomous aldosterone secretion and exists on a spectrum ranging from unilateral to bilateral PA. Unilateral PA includes APAs and unilateral adrenal hyperplasia. For bilateral PA, the most common cause is bilateral idiopathic hyperaldosteronism (IHA).^109^

Primary aldosteronism prevalence is about 5%-20% in hypertensive patients, depending on the severity of hypertension and the population studied.^109-113^ In contrast to primary adrenal cortisol excess, there is not a clear-cut sex difference in PA prevalence although recent data suggest that PA may be slightly more prevalent in men.^114-117^ Some studies showed that bilateral PA was more common in women,^118-120^ but this was not confirmed by others.^121-123^ A Japanese multicentre study including 2122 patients with PA found that women over the age of 50 were more likely to present with bilateral PA than younger counterparts, while an opposite trend was observed in men where unilateral PA was more frequent after the age of 50.^118^ The authors proposed that the high proportion of unilateral PA in younger women may be explained by *KCNJ5* mutations, the most common somatic mutation observed in APAs. A recent study from China including 482 patients who underwent successful adrenal vein sampling to differentiate unilateral from bilateral PA, found that imaging appearances of the adrenal glands, coupled with serum potassium levels, were more strongly predictive of unilateral PA in men compared to women.^124^

Somatic mutations, including *KCNJ5*, *ATP1A1*, *ATP2B3*, *CACNA1D*, *CACNA1H*, *CLCN2*, and *CTNNB1* have been discovered in more than 90% of APAs.^125,126^ The first 6 mutations trigger aldosterone secretion by activating the intracellular calcium signalling pathway, while *CTNNB1* encodes β-catenin, a protein in the Wnt/β-catenin signalling pathway, involved in adrenal cortex maintenance, adrenal steroidogenesis, and adrenal tumourigenesis.^7^ The constitutive activation of β-catenin in mouse models caused ZG hyperplasia and hyperaldosteronism. Recently, new somatic mutations, *CADM1*, interfering with gap junction communication, and *SLC30A1*, probably causing abnormal ion transport, have been described.^127,128^

*KCNJ5* accounts for 40%-70% of APA cases,^125,126,129,130^ except in Blacks, where *CACNA1D* is the more prevalent at 42% (vs *KCNJ5* at 34%).^131^ The highest prevalence of *KCNJ5* mutations is found in Asian populations.^132-136^ Patients with somatic *KCNJ5* mutation are typically younger women (1.6-4.4:1 female-to-male ratio), with higher aldosterone and hybrid steroid excretion due to co-expression of CYP11B1 and CYP11B2 in APA tissue, larger tumour size, and better clinical outcomes after adrenalectomy (Table 1).^125,129,137-139^

Activating *CTNNB1* mutations are also more common in women than men (female-to-male ratio 2:1).^125,140,141^ Data are, however, limited due to the low prevalence of *CTNNB1* mutations in APAs (2%-5% of cases).^140-142^ In humans, 1 case series described the potential link between *CTNNB1* mutation with LHCGR and Gonadotropin-Releasing Hormone (GnRH) receptors in 2 pregnant patients and 1 post-menopausal patient with PA.^143^ A further study has revealed that co-existing mutations of *CTNNB1* with *GNA11* or *GNAQ* may elucidate the link between LHCGR expression and PA.^144^  *GNA11* and *GNAQ* encode G-protein α subunits G11 and Gq, respectively. Mutation in these genes enhances guanosine triphosphate (GTP) activity, stimulating aldosterone production. Notably, *GNA11* or *GNAQ* mutations alone are clinically silent. In a cohort of patients from the UK and Ireland with co-existing *CTNNB1*, *GNA11*, or *GNAQ* mutations, 9 patients (8 women, 1 man) developed PA during puberty, pregnancy, or menopause, and their LHCGR expression was more than 10-fold higher compared to *CTNNB1*-negative APAs. However, a study from another group found no *CTNNB1* mutations in 11 patients with GnRH-responsive aldosterone secretion.^145^

There are also sex- and subtype-specific differences in the cardiometabolic outcomes of patients with PA. In a large Japanese study, men with PA were found to have a more severe phenotype than women, including higher BMI, longer duration of hypertension, lower glomerular filtration rate, higher anti-hypertensive medication requirements, and hypokalaemia.^118^ Men with PA, both unilateral and bilateral, had higher BMI and visceral adiposity than normotensive matched controls, while this was the case only in women with IHA.^146^ Women with IHA were also more likely to be obese^118^ and to have type 2 diabetes and dyslipidaemia than those with unilateral PA.^147^ A higher risk of kidney function decline after spironolactone initiation was also observed in women with IHA compared to men.^148^ Notably, however, women with APAs are more likely to have complete clinical success after unilateral adrenalectomy, defined mainly by the resolution of hypertension.^149-154^ This could be partly driven by the higher prevalence of *KCNJ5* mutations in women, the protective cardiovascular function of oestrogens, and the potentially dysfunctional oestrogen signalling in APA tissue.^29,155,156^

## Conclusions

Adrenal CS and somatic mutations of cAMP-related genes in CPAs are significantly more prevalent in females. Women with adrenal incidentalomas are more likely to have MACS and appear to be more susceptible to the detrimental effects of cortisol excess. Men with PA tend to have a worse cardiometabolic risk profile than women. In APAs, women have a higher prevalence of *KCNJ5* mutations than men and are more likely to have better clinical outcomes after adrenalectomy, but they carry a higher risk of renal function deterioration after starting mineralocorticoid receptor antagonists. Such sex-specific considerations are important in the care of patients diagnosed with BATs and should guide management. The mechanisms underlying the observed sexual dimorphism in BAT development, manifestation, and prognosis remain to be fully elucidated.
